# Brief Version of Caffeine Expectancy Questionnaire in Brazil

**DOI:** 10.3389/fnut.2021.695385

**Published:** 2021-06-30

**Authors:** Guilherme Falcão Mendes, Caio Eduardo Gonçalves Reis, Eduardo Yoshio Nakano, Renata Puppin Zandonadi

**Affiliations:** ^1^Department of Nutrition, School of Health Sciences, University of Brasilia (UnB), Brasilia, Brazil; ^2^Department of Statistics, Central Institute of Sciences, University of Brasilia (UnB), Brasilia, Brazil

**Keywords:** short, brief, questionnaire, assessment, caffeine, expectancy, Brazilian-Portuguese

## Abstract

The use of extensive questionnaires has the limitation of filling time bias, related to the ability to focus and accurately respond to many items, justifying the necessity for a brief version. This study aimed to build a brief version of the Caffeine Expectancy Questionnaire in Brazil (B-CaffEQ-BR) composed of 21 items divided into seven factors, with as adequate consistency and reproducibility as the full version. Quantitative procedures using statistical modeling were applied using the CaffEQ-BR (full version) database keeping the Mean Absolute Error (MAE) (based on the full version) <0.5 and Cronbach's α and Intraclass Correlation Coefficient (ICC) ≥0.7. The expert panel (*n* = 3), in a blind design, evaluated the semantic structuring within the options indicated by previous statistical modeling until the agreement of the expert panel. The participants (*n* = 62), Brazilian adults who were regular caffeine consumers (175.8 ± 94.4 mg/day), of whom 62.9% were women, 33.1 ± 9.7 years, 24.5 ± 3.8 kg/m^2^, and 62.9% of whom self-identified as white, were asked to respond twice to the online questionnaire in 48–72 h. The first sample (*n* = 40) tested interobserver reproducibility with the double application of B-CaffEQ-BR. Another sample (*n* = 22) answered the CaffEQ-BR (full version) and B-CaffEQ-BR, and the last sample (*n* = 18) performed the reverse process. The B-CaffEQ-BR presented excellent internal consistency (Cronbach's α ≥ 0.729) and overall reproducibility (ICC ≥ 0.915) for the entire questionnaire and its seven factors. The B-CaffEQ-BR can be a valuable tool in caffeine research with the Brazilian adult population.

## Introduction

Caffeine is the most consumed psychoactive substance globally (1), used mainly in coffee, which forms a part of Brazilian eating habits (2–4). In addition, caffeine is the most common active ingredient in coffee, mate, guarana, green tea, cocoa, and its derivatives (5). Caffeine is widely studied and presents several guidelines for use, dosage, and safe consumer limits (<400 mg/day) (6–8). The intake of products containing caffeine is associated with its taste characteristics, population eating habits, and the expectations related to the effects of caffeine on the body (i.e., physiological and performance aspects) (9).

Studies confirm that the expected effects of caffeine play a subjective role in the belief around its consumption (10–12). Thus, the expectations associated with caffeine consumption and its effects can play an essential role in the development, maintenance, and reinforcement of their consumption showing the importance of knowledge about the subjective perceptions of caffeine consumption (9, 12, 13). In this regard, validated questionnaires to assess caffeine expectancy have been recently published to understand the expectations of caffeine effects (i.e., mood, appetite, sleep/wakefulness, physical performance, and other factors) and consumption (9, 13–15).

Chronologically, Heinz et al. (12) developed the Caffeine Expectation Questionnaire (CEQ) composed of 37 items distributed in four factors that represent (i) “withdrawal symptoms,” (ii) “positive effects,” (iii) “acute negative effects,” and (iv) “mood effects.” Subsequently, Huntley and Juliano (14) developed the Caffeine Expectancy Questionnaire (CaffEQ), which consists of 47 items divided into seven factors: (i) “addiction,” (ii) “energy and mental activity,” (iii) “appetite suppression,” (iv) “improved mood/sociability,” (v) “improved physical performance,” (vi) “anxiety and negative effects,” and (vii) “sleep disorders.” In the process of translation and cultural validation, Schott et al. (15) validated the CaffEQ for the adult German-speaking population (Germany, Austria, and Switzerland). More recently, Kearns et al. (13) proposed a brief English version of CaffEQ validated in a sample (*n* = 975) of undergraduate adult students from the United States of America (US). They reduced the questionnaire from 47 to 20 items preserving the original seven factors, with satisfactory internal and external consistency, stating that the use of short questionnaires is better accepted by the public, especially in the context of a self-applied questionnaire.

Our group recently published the Caffeine Expectancy Questionnaire in Brazil (CaffEQ-BR) (16) with semantic translation and cultural validation to Brazilian–Portuguese language based on the wide application covering all the states of the Brazilian territory (*n* = 4,202). CaffEQ-BR presented satisfactory consistency and reproducibility as the original version (CaffEQ), maintaining the seven factors and the 47 items. One of the difficulties presented in our CaffEQ-BR study was to apply a lengthy questionnaire in a large population sample, with a frequent complaint of repetitive items and the long time required for attentive completion (16). The time to complete a questionnaire (due to the number of items and volume of text/words) is inversely associated with the response rate and accuracy. The length of the questionnaire (47 items) may hinder the widespread application of this tool (17). Therefore, brief versions of questionnaires validated to specific populations are emphasized to save time and resources, and to increase the adherence of participants to the studies. Hence, the development of the brief version of the Caffeine Expectancy Questionnaire in Brazil (B-CaffEQ-BR) is justified given the current context of online self-filling application questionnaires having become a worldwide trend (18).

Smith et al. (19) reported that some questions must be considered when developing a brief version of the questionnaire: (i) developing the brief version of a previously sufficiently validated full version; (ii) showing that the brief version preserves the coverage of the content of each factor and the scale of each factor is measured in the same way; (iii) showing that the brief version has an adequate variety of equivalent overlap and the ability to reproduce the factorial structure; (iv) showing that each factor in the brief form is valid in an independent sample, with similar or better consistency indexes than the full version; and (v) showing that the brief version offers significant savings in time and/or resources. All these assumptions were fully observed in the construction of the B-CaffEQ-BR.

Even with the brief English CaffEQ (13) questionnaire previously available in the literature, it is important to emphasize that the translation and cultural validation of an instrument in the brief version does not guarantee the achievement of the same result based on the full version (14). For this reason, we initially translated and validated the full version of CaffEQ-BR (16) into Portuguese and Brazilian culture. Therefore, it was more appropriate to construct the B-CaffEQ-BR version based on the data obtained in the full version (CaffEQ-BR) with the treatment of construct from a qualitative and quantitative methodological perspective.

Therefore, this study aimed to validate the B-CaffEQ-BR. We hope this study can provide a questionnaire with as satisfactory internal and external validation as the full CaffEQ-BR version. The brief version aims to have a similar ability to characterize according to the expectations of caffeine use in the Brazilian adult population, with more straightforward application in large populations, which can be useful in future caffeine studies.

## Materials and Methods

The present study used the dataset from the full version CaffEQ-BR, previously validated in the Brazilian–Portuguese language (16). The CaffEQ-BR consists of seven factors and 47 items, assessed using a six-point Likert scale. For the construction of the B-CaffEQ-BR, the present study was carried out in four stages: (1) Quantitative evaluation by statistical modeling, (2) Qualitative assessment of semantic structure by the panel of experts, (3) Internal consistency and reproducibility analysis, and (4) Survey of full and brief versions by the mixed two-way model.

The study was approved by the Ethics Committee of the Catholic University of Brasília (Brasília, Brazil) (number: 23019319.3.0000.0029) and followed the guidelines established by the Declaration of Helsinki. The volunteers were informed about the study protocol and provided web-based consent. The research was conducted using a web platform, Google Forms™ (20). The online form maintained the layout, content, and the general instructions contained in the original version of CaffEQ-BR, in addition to self-reported identification data and average weekly consumption of caffeine sources as described in detail by Mendes et al. (16).

### Quantitative Evaluation by Statistical Modeling

For the quantitative analysis, from the preliminary information obtained from the database of full CaffEQ-BR (*n* = 4,202), it was possible to obtain all combinations of three items by a factor whose present Mean Absolute Error (MAE) (21) is <0.5 and internal alpha consistency of Cronbach's α ≥ 0.7 (22). This previous procedure was paramount to providing a ranking of options for the subsequent semantic analysis of the specialist panel. The objective of this stage before the qualitative assessment was to reduce the structure of the full CaffEQ-BR (seven factors/47 items) (16) for a brief version with seven factors and 21 items (three items per factor).

### Qualitative Assessment of Semantic Structure by the Expert Panel

In the qualitative analysis, an expert panel (*n* = 3) assessed the semantic relevance of items from the ranking indicated by the quantitative analysis. The funneling process (in a blind design) was carried out in several stages when the experts unanimously agreed to maintain or delete the item until three items per factor remained. Finally, the brief version selected and approved by the quantitative and qualitative analysis (seven factors/21 items) was applied to a convenience sample of Brazilian adults living in Brazil to assess the internal consistency and reproducibility.

### Internal Consistency and Reproducibility

The internal consistency and reproducibility of the B-CaffEQ-BR were analyzed using a convenience sample of Brazilian adults (*n* = 40) who were regular caffeine consumers (188.7 ± 106.5 mg/day) and who never had contact with the questionnaire. They answered the B-CaffEQ-BR twice (test and retest) within 48–72 h intervals (16, 23). These data were applied to analyze internal consistency and reproducibility due to the convergence of responses in the test and retest (24).

### Survey of Full and Brief Version by the Mixed Two-Way Model

The composition and psychometric properties of the brief version were compared with full CaffEQ-BR. Therefore, another convenience sample (*n* = 40) of regular caffeine consumers (160.0 ± 71.8 mg/day) was recruited in which half of the participants (*n* = 20) first filled out the full CaffEQ-BR and after 48–72 h (16, 23), they were asked to fill in the B-CaffEQ-BR. The other half did the reverse process, initially answering the B-CaffEQ-BR and then the full CaffEQ-BR (within 48–72 h interval). This method aims to determine whether there was interference from the “learning bias” issue. According to Smith et al. (19), this methodological care reinforces the quality and applicability of the construct.

### Statistical Analysis

The MAE test was used to perform the qualitative analysis, representing the average divergence of the brief and scores of the full versions, with the zero-value indicating perfect agreement. The condition of MAE <0.5 means that, on average, this divergence is <0.5 (i.e., 10% error on a five-point scale) (21).

The reproducibility of the questionnaire between the test and retest was analyzed by the Intraclass Correlation Coefficient (ICC). The absolute agreement was used to determine ICC, considering the average agreement of the two applications. According to Cicchetti (25), an excellent ICC agreement between evaluators is considered when the value ≥0.75 and good agreement is between 0.74 and 0.60. Cronbach's α was used to check the internal consistency of questionnaire factors, where values ≥0.7 indicate that the factors are consistent (22). The agreement of the B-CaffEQ-BR scores compared to the full version was assessed using the ICC (absolute agreement) obtained through a mixed two-way model.

All tests were performed considering a significance level of 5%, using the statistical packages IBM SPSS (Statistical Package for Social Sciences) version 22 (IBM SPSS Statistics for Windows, IBM Corp, Armonk, NY, US) and IBM SPSS AMOS (Analysis of Moment Structures) version 22 (Amos, IBM SPSS, Chicago, IL, US).

## Results

**Figure 1:**
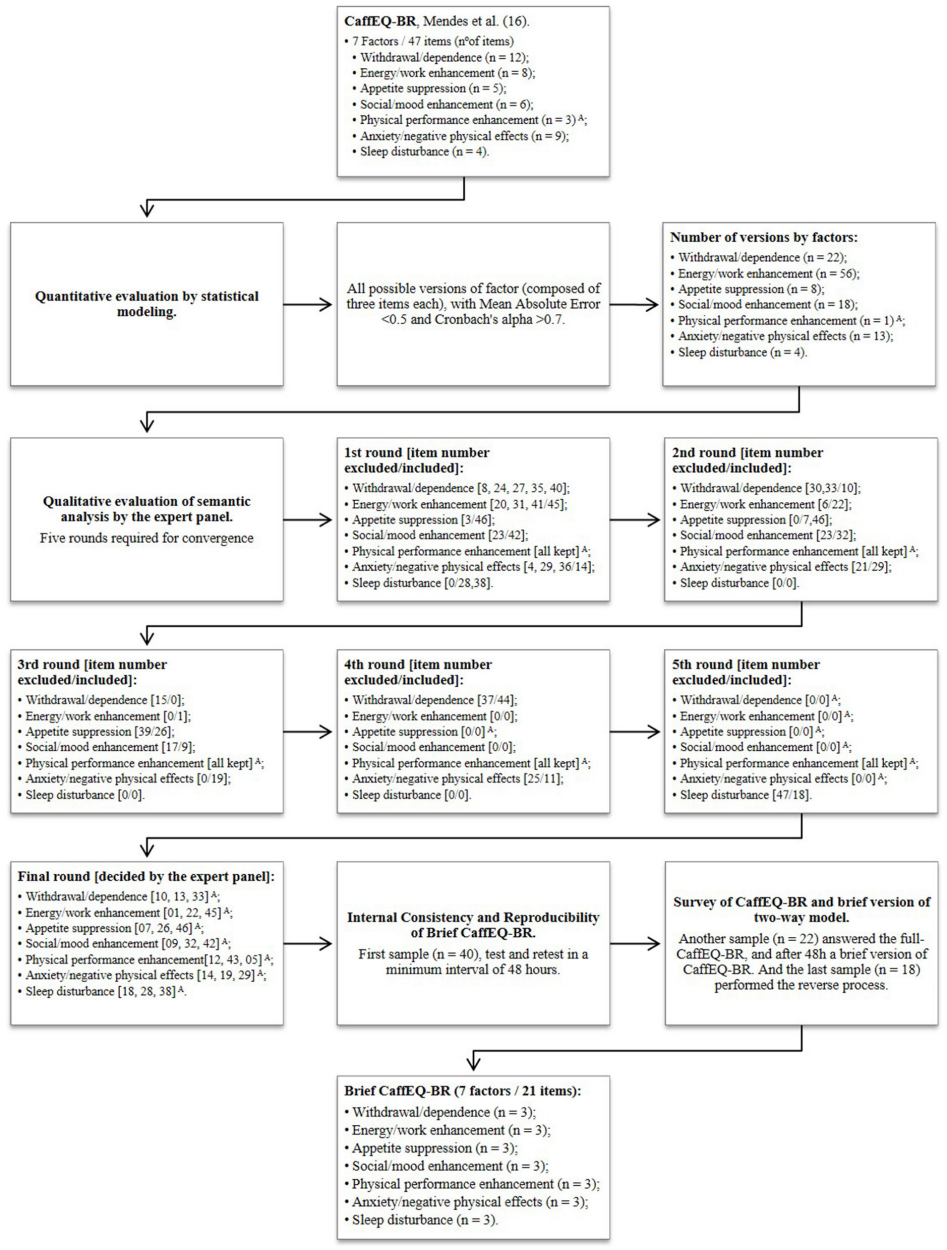
Process stages of the brief version of Brazilian Caffeine Expectancy Questionnaire in Brazil (B-CaffEQ-BR). ^A^Three items are defined per factor. In this case, the factor Physical performance enhancement has only three items, so it was not reduced; Note: Number zero [0] indicates no change.

In Figure 1, the flowchart shows the results obtained with the multiple steps described in the methods. The results present convergence of the quantitative analysis results by statistical modeling and semantic agreement obtained by the expert panel. The B-CaffEQ-BR was performed with a convenience sample of 62 Brazilian adult usual consumers of caffeine (175.8 ± 94.4 mg/day). The sample profile composed of 62.9% females, 33.1 ± 9.7 years, body mass index 24.5 ± 3.8 kg/m^2^, self-identified as white 62.9%, 80.6% without a diagnosis of chronic diseases, 71% physically active (>150 weekly min of physical exercise), 53.2% with postgraduate education, and 40.3% with average monthly family income from BRL 10,001.00 to 20,000.00 (BRL 5.76 = USD 1.00) on the last day of data collection, October 2020.

### Internal Consistency of B-CaffEQ-BR

Table 1 shows the internal consistency of Cronbach's α values (α ≥ 0.729) for all seven factors and the entire questionnaire (α = 0.906). These results were obtained with a 55.3% reduction of the questionnaire, from 47 to 21 items.

**Table 1:** Internal consistency of the brief version of Caffeine Expectancy Questionnaire in Brazil (B-CaffEQ-BR) (*n* = 62^A^).

**Factors**	**N. items**	**Cronbach's α**
Withdrawal/dependence	3	0.729
Energy/work enhancement	3	0.799
Appetite suppression	3	0.819
Social/mood enhancement	3	0.774
Physical performance enhancement	3	0.909
Anxiety/negative physical effects	3	0.837
Sleep disturbance	3	0.859
**Entire questionnaire**	**21**	**0.906**

A *n = 62 participants in total, regular adult caffeine consumers in Brazil (175.8 ± 94.4 mg/day). Subsamples: n = 18 brief to brief to full + n = 22 only brief to brief + n = 22 full to brief*.

### Reproducibility and Agreement of CaffEQ-BR Versions

The overall reproducibility showed an excellent ICC agreement (brief–brief = 0.978 and full–brief = 0.920) for the entire questionnaire. Regardless of the order of the applications of questionnaires (brief–brief or full–brief) the ICC values are excellent (≥0.780) for all seven factors, except for “anxiety/negative physical effects” on full x brief analysis (ICC = 0.726; good agreement) (Table 2). These findings confirm the agreement between the two versions of CaffEQ-BR.

**Table 2:** Mean (SD) of Caffeine Expectancy Questionnaire in Brazil (CaffEQ-BR) full and brief scores and measure of agreement.

	**Brief** **×** **Brief (*****n*** **=** **40)^A^**			**Full** **×** **Brief (*****n*** **=** **40)^A^**		
**Factors (n^**°**^ items Full to Brief)**	**Brief 1 Mean (SD)**	**Brief 2 Mean (SD)**	**ICC^B^**	**Full Mean (SD)**	**Brief Mean (SD)**	**ICC^B^**
Withdrawal/dependence (12 to 3)	3.04 (1.49)	2.98 (1.50)	0.953	2.55 (1.50)	2.69 (1.37)	0.931
Energy/work enhancement (8 to 3)	4.07 (1.36)	4.09 (1.49)	0.920	3.90 (1.36)	4.26 (1.30)	0.829
Appetite suppression (5 to 3)	2.12 (1.37)	2.16 (1.41)	0.975	1.82 (1.19)	1.69 (1.07)	0.908
Social/mood enhancement (6 to 3)	3.19 (1.47)	3.26 (1.45)	0.949	3.07 (1.37)	2.89 (1.27)	0.780
Physical performance enhancement (3 to 3)	3.81 (1.50)	3.87 (1.65)	0.960	3.45 (1.57)	3.90 (1.57)	0.885
Anxiety/negative physical effects (9 to 3)	1.94 (1.08)	1.95 (1.25)	0.915	1.48 (0.66)	1.86 (0.76)	0.726
Sleep disturbance (4 to 3)	3.31 (1.62)	3.08 (1.62)	0.946	2.88 (1.65)	2.65 (1.69)	0.882
**Entire questionnaire**	**3.07 (0.97)**	**3.05 (0.98)**	**0.978**	**2.74 (0.90)**	**2.79 (0.80)**	**0.920**

A *n = 18 brief–full and 22 full–brief subsample, regular adult caffeine consumers in Brazil (160.0 ± 71.8 mg/day)*;

b *ICC, Intraclass Correlation Coefficient (absolute agreement) obtained through a mixed two-way model; Likert scale from 1 = Very unlikely to 6 = Very likely*.

## Discussion

The B-CaffEQ-BR, a brief version of the questionnaire, showed excellent reproducibility and adequate internal consistency, similar to the full CaffEQ-BR version (*n* = 4,202; α = 0.948; and ICC = 0.976) as previously published (16). These findings confirm that the convenience sample of Brazilian adult habitual caffeine consumers was sufficient to verify the reproducibility and internal consistency of B-CaffEQ-BR. The expectations of the caffeine effects were similar across all seven factors of B-CaffEQ-BR compared to the full version (16).

In addition, B-CaffEQ-BR obtained a significant reduction from 47 to 21 items, presenting a real time saver for completion (reduction from ~10 to 5 min) (internal data). This substantial saving of time is important to popularize online surveys (18). Therefore, the B-CaffEQ-BR fits better to web-based research demands, and the current model is widely used through the internet and social networks (13, 15, 16, 20). The questionnaire is semantically adapted to the Brazilian language and culture and can be applied to the Brazilian adult population of habitual caffeine consumers. This instrument is essential for the proper screening of research participants around caffeine studies (26). Sometimes, sample heterogeneity can compromise the internal or external validation of the study (27). The sample profile regarding the expectations of caffeine effects reduces the possible confounding variables in the analysis (26, 28).

The application of a methodological and statistical approach allowed the reduction of the questionnaire resulting in excellent internal consistency and overall reproducibility for the entire questionnaire and its seven factors (Tables 1, 2). The possibility of using the robust database from the full CaffEQ-BR allowed a targeted selection of the 21 items of the brief version. Thereby, this study was able to achieve reproducibility and internal consistency of the brief version without the intention of assessing the expectations of caffeine effects through the B-CaffEQ-BR.

Regarding Table 1, the internal consistency of the entire questionnaire is not the average of the factors, but Cronbach's α scores considering the whole instrument. The isolated factors presented a satisfactory Cronbach's α (α ≥ 0.729), and the entire questionnaire showed a very satisfactory internal consistency (α = 0.906). It reinforces the need for factor analysis of the entire questionnaire (22). Another important aspect was the semantic evaluation of the panel of experts. In addition to the statistical approach, semantic analyses were performed by funneling process to reduce from 47 to 21 items with the seven factors preserved. The brief English version of the CaffEQ also presented a similar reduction from 47 to 20 items keeping the seven factors and showed a satisfactory internal consistency (*n* = 975; α = 0.93) (13), similar to the original CaffEQ version (*n* = 1,046; α = 0.96) (14).

Concerning the composition of the B-CaffEQ-BR (21 items) compared with the brief English version (20 items) (13), we found that 11 items (47.6%) converge between versions (items: 1, 7, 12, 13, 19, 26, 28, 42, 43, 45, and 46). The English and Portuguese–Brazilian versions are different, not only in linguistic and cultural adaptation but also in the selected items in the questionnaire. Therefore, they must be applied to their respective populations, for which they were validated.

The adoption of the mixed two-way model was essential to confirm that, regardless of the order of application (brief–brief or brief–full), the instrument presented an excellent overall reproducibility (ICC = 0.978 and 0.920, respectively) (Table 2), of the full version (ICC = 0.976) (16). This methodological strategy is an additional precaution to control the learning bias since the time interval between tests (48–72 h) should solve this issue. The inversion application order of the questionnaires can avoid this memorization/learning risk of bias (29).

A relevant result obtained was the high mean value (3.90–4.26) observed in the “Energy/work enhancement” factor showing a probable effect on the expectation of stimulating caffeine effects by the responders. The opposite (improbable effect) was observed for the “Anxiety factor/negative physical effects” factor, which presented a mean value <2.0. In all studies that applied the CaffEQ, the samples were composed of regular caffeine consumers (13–16). People who presented negative effects after caffeine consumption are probably not regular consumers (11, 14). Therefore, a tendency to express more positive than negative effects is expected (14, 30). Many people possibly scored high value for “Energy/work enhancement” due to the expectation of productivity at work and studies, a fact more observed in Western consumers (30), such as the Brazilian adults participating in the present study. Other studies show high consumption of caffeine sources above the population average in specific groups, such as University students (31, 32) and men of working age at work (3, 31). In any case, our sample is similar on the sociodemographic aspects, caffeine consumption, and expectancy recorded in the CaffEQ-BR full version (16).

The Caffeine Expectancy Questionnaire in Brazil, in full and brief versions, aims to discover individual caffeine expectancy. However, the benefits of the brief version are accuracy (as well as the full version) and quick and easy application. In the sporting context, performance-related factors have more relevance (e.g., “energy/work enhancement,” “social/mood enhancement,” “physical performance enhancement,” and “anxiety/negative physical effects”). Understanding these expectations is critical to assess the risk of bias in clinical trials in sports science due to the ergogenic or ergolytic effects of placebo associated with the effects of caffeine. Literature shows how the placebo effect (33, 34), either due to excess or lack of expectation about the effect of caffeine (12, 14, 35, 36), can represent a possible risk of bias for the main findings of the research. For example, scores above the national average for factors may indicate a tendency to respond to caffeine supplementation. In the comparison of the average results in Table 2, with the averages observed nationally (16), by factors: withdrawal/dependence, averages between 2.55 and 3.04, national 3.48 (1.43); energy/work enhancement, between 3.90 and 4.26, national 4.14 (1.32); appetite suppression, between 1.69 and 2.16, national 2.24 (1.17); social/mood enhancement, between 2.89 and 3.26, national 3.41 (1.38); physical performance enhancement, between 3.45 and 3.90, national 3.47 (1.51); anxiety/negative physical effects, between 1.48 and 1.95, national 1.78 (0.77); sleep disturbance, between 2.65 and 3.31, national 2.47 (1.62). Moreover, its application in clinical practice, to know the caffeine expectancy profile, can help to adjust the caffeine prescription. However, further studies are necessary to evaluate other applications of CaffEQ-BR with more specific purposes.

Some limitations of the present study must be observed. Web-based research has an inherent selection bias, limited to those with access to computers and internet resources, a fact described and observed in other studies with CaffEQ (13–16). Web-based research is limited to control environmental factors during the research application that may add some risk of bias to the data collected. However, the robustness and consistency of our results suggest that respondents answered the questions consciously. A fact observed in the present study is that the average daily self-reported caffeine intake (175.8 ± 94.4 mg/day) was lower than that observed in the full CaffEQ-BR (265 ± 159 mg/day) (16) and slightly higher than Brazil nationwide estimation (115 ± 96 mg/day) (37). This provides indirect evidence that our sample is regular caffeine consumers with a weekly and daily average that may reflect the consumption profile of the general population.

## Conclusion

The B-CaffEQ-BR is available for the Brazilian adult population. This study provides a reliability questionnaire, expressed by adequate internal consistency and reproducibility, similar to the full version of the CaffEQ-BR. The brief version can characterize the expectations of the effect of caffeine on adult Brazilian consumers, with more straightforward and feasible online applications in large populations, and may be helpful in future studies on caffeine.

## Data Availability Statement

The original contributions generated for this study are included in the article/Supplementary Material, further inquiries can be directed to the corresponding author/s.

## Ethics Statement

The studies involving human participants were reviewed and approved by the Ethics Committee of the University Católica of Brasília (Brasília, Brazil) (number: 23019319.3.0000.0029) and followed the guidelines established by the Declaration of Helsinki. The volunteers were informed about the study protocol and provided web-based consent. The patients/participants provided their written informed consent to participate in this study.

## Author Contributions

GM, CR, and RZ: Conceptualization. GM, CR, EN, and RZ: methodology, validation, writing, review, and editing. GM and EN: software and data curation. EN: formal analysis. GM: investigation and writing the original draft preparation. CR and RZ: resources and funding acquisition. GM and RZ: visualization. RZ: supervision. GM and CR: project administration. All the authors have read and agreed to the published version of the manuscript.

## Conflict of Interest

The authors declare that the research was conducted in the absence of any commercial or financial relationships that could be construed as a potential conflict of interest.

## References

[1] Reyes CM Cornelis MC. Caffeine in the diet: Country-level consumption and guidelines. Nutrients. (2018) 10:1772. 10.3390/nu10111772 30445721 PMC6266969

[2] Souza A de M Pereira RA Yokoo EM Levy RB Sichieri R. Most consumed foods in Brazil: national dietary survey 2008-2009. Rev Saude Publica. (2013) 47:190s−9s. 10.1590/s0034-89102013000700005 23703263

[3] Sousa AG Da Costa THM. Usual coffee intake in Brazil: results from the national dietary survey 2008-9. Br J Nutr. (2015) 113:1615–20. 10.1017/S0007114515000835 25851731

[4] Pereira RA Souza AM Duffey KJ Sichieri R Popkin BM. Beverage consumption in Brazil: results from the first national dietary survey. Public Health Nutr. (2015) 18:1164–72. 10.1017/S1368980014001657 25158687 PMC4344434

[5] Heckman MA Weil J de Mejia EG. Caffeine (1, 3, 7-trimethylxanthine) in foods: a comprehensive review on consumption, functionality, safety, and regulatory matters. J Food Sci. (2010) 75:R77–87. 10.1111/j.1750-3841.2010.01561.x 20492310

[6] Maughan RJ Burke LM Dvorak J Larson-Meyer DE Peeling P Phillips SM. IOC consensus statement: dietary supplements and the high-performance athlete. Br J Sports Med. (2018) 52:439–55. 10.1136/bjsports-2018-099027 29540367 PMC5867441

[7] Wikoff D Welsh BT Henderson R Brorby GP Britt J Myers E. Systematic review of the potential adverse effects of caffeine consumption in healthy adults, pregnant women, adolescents, and children. Food Chem Toxicol. (2017) 109:585–648. 10.1016/j.fct.2017.04.002 28438661

[8] Poole R Kennedy OJ Roderick P Fallowfield JA Hayes PC Parkes J. Coffee consumption and health: umbrella review of meta-analyses of multiple health outcomes. BMJ. (2017) 359:j5024. 10.1136/bmj.j5024 29167102 PMC5696634

[9] Ágoston C Urbán R Király O Griffiths MD Rogers PJ Demetrovics Z. Why do you drink caffeine? The development of the motives for caffeine consumption questionnaire (MCCQ) and its relationship with gender, age and the types of caffeinated beverages. Int J Ment Health Addict. (2018) 16:981–99. 10.1007/s11469-017-9822-3 30147634 PMC6096549

[10] Shabir A Hooton A Tallis J Higgins MF. The influence of caffeine expectancies on sport, exercise, and cognitive performance. Nutrients. (2018) 10:1528. 10.3390/nu10101528 30336606 PMC6212857

[11] Dömötör Z Szemerszky R Köteles F. Subjective and objective effects of coffee consumption - caffeine or expectations? Acta Physiol Hung. (2015) 102:77–85. 10.1556/APhysiol.101.2014.012 25481367

[12] Heinz AJ Kassel JD Smith EV. Caffeine expectancy: instrument development in the rasch measurement framework. Psychol Addict Behav. (2009) 33:500–11. 10.1037/t00052-000 19769434

[13] Kearns NT Blumenthal H Natesan P Zamboanga BL Ham LS Cloutier RM. Development and initial psychometric validation of the brief-caffeine expectancy questionnaire (B-CaffEQ). Psychol Assess. (2018) 30:1597–611. 10.1037/t68055-000 29927303 PMC6852668

[14] Huntley ED Juliano LM. Caffeine Expectancy Questionnaire (CaffEQ): construction, psychometric properties, and associations with caffeine use, caffeine dependence, and other related variables. Psychol Assess. (2012) 24:592–607. 10.1037/a0026417 22149323

[15] Schott M Beiglböck W Neuendorff R. Translation and validation of the Caffeine Expectancy Questionnaire (CaffEQ). Int J Ment Health Addict. (2016). 14:514–25. 10.1037/t56049-00032731330

[16] Mendes GF Reis CEG Nakano EY da Costa THM Saunders B Zandonadi RP. Translation and validation of the Caffeine Expectancy Questionnaire in Brazil (CaffEQ-BR). Nutrients. (2020) 12:2248. 10.3390/nu12082248 32731330 PMC7468745

[17] Rolstad S Adler J Rydén A. Response burden and questionnaire length: is shorter better? A review and meta-analysis. Value Heal. (2011) 14:1101–8. 10.1016/j.jval.2011.06.003 22152180

[18] Herrero J Meneses J. Short Web-based versions of the perceived stress (PSS) and Center for Epidemiological Studies-Depression (CESD) Scales: a comparison to pencil and paper responses among Internet users. Comput Human Behav. (2006) 22:830–46. 10.1016/j.chb.2004.03.007

[19] Smith GT McCarthy DM Anderson KG. On the sins of short-form development. Psychol Assess. (2000) 12:102–11. 10.1037/1040-3590.12.1.102 10752369

[20] Knapp H Kirk SA. Using pencil and paper, Internet and touch-tone phones for self-administered surveys: does methodology matter? Comput Human Behav. (2003) 19:117–34. 10.1016/S0747-5632(02)00008-0

[21] Willmott CJ Matsuura K. Advantages of the mean absolute error (MAE) over the root mean square error (RMSE) in assessing average model performance. Clim Res. (2005) 30:79–82. 10.3354/cr030079

[22] Streiner DL Streiner DL. Starting at the beginning: an introduction to coefficient alpha and internal consistency. J Pers Assess. (2016) 80:99–103. 12584072 10.1207/S15327752JPA8001_18

[23] Hargreaves SM Nakano EY Zandonadi RP. Brazilian vegetarian population—influence of type of diet, motivation and sociodemographic variables on quality of life measured by specific tool (VEGQOL). Nutrients. (2020) 12:1406. 10.3390/nu12051406 32422862 PMC7284834

[24] Meijering JV Kampen JK Tobi H. Quantifying the development of agreement among experts in Delphi studies. Technol Forecast Soc Change. (2013) 80:1607–14. 10.1016/j.techfore.2013.01.003

[25] Cicchetti DV. Guidelines, criteria, and rules of thumb for evaluating normed and standardized assessment instruments in psychology. Psychol Assess. (1994) 6:284. 10.1037/1040-3590.6.4.284

[26] Ágoston C Urbán R Richman MJ Demetrovics Z. Caffeine use disorder: An item-response theory analysis of proposed DSM-5 criteria. Addict Behav. (2018) 81:109–16. 10.1016/j.addbeh.2018.02.012 29454178

[27] Watson PF Petrie A. Method agreement analysis: a review of correct methodology. Theriogenology. (2010) 73:1167–79. 10.1016/j.theriogenology.2010.01.003 20138353

[28] Irons JG Bassett DT Prendergast CO Landrum RE Heinz AJ. Development and initial validation of the caffeine consumption questionnaire-revised. J Caffeine Res. (2016) 6:20–5. 10.1089/jcr.2015.0012

[29] Kaplan RM Saccuzzo DP. Psychological Testing: Principles, Applications, and Issues. 5th ed. Pacific Grove, CA: Brooks, (2001).

[30] Chan EY Maglio SJ. Coffee cues elevate arousal and reduce level of construal. Conscious Cogn. (2019) 70:57–69. 10.1016/j.concog.2019.02.007 30849742

[31] Dillon P Kelpin S Kendler K Thacker L Dick D Svikis D. Gender differences in any-source caffeine and energy drink use and associated adverse health behaviors. J Caffeine Adenosine Res. (2019) 9:12–9. 10.1089/caff.2018.0008 30944911 PMC6444914

[32] Mahoney CR Giles GE Marriott BP Judelson DA Glickman EL Geiselman PJ. Intake of caffeine from all sources and reasons for use by college students. Clin Nutr. (2019) 38:668–75. 10.1016/j.clnu.2018.04.004 29680166

[33] Saunders B de Oliveira LF da Silva RP de Salles Painelli V Gonçalves LS Yamaguchi G. Placebo in sports nutrition: a proof-of-principle study involving caffeine supplementation. Scand J Med Sci Sport. (2017) 27:1240–7. 10.1111/sms.12793 27882605

[34] Saunders B Saito T Klosterhoff R de Oliveira LF Barreto G Perim P. “I put it in my head that the supplement would help me”: open-placebo improves exercise performance in female cyclists. PLoS ONE. (2019) 14:e0222982. 10.1371/journal.pone.0222982 31550286 PMC6759201

[35] Grgic J. Are there non-responders to the ergogenic effects of caffeine ingestion on exercise performance? Nutrients. (2018) 10:1736. 10.3390/nu10111736 30424511 PMC6267019

[36] Del Coso J Lara B Ruiz-Moreno C Salinero JJ. Challenging the myth of non-response to the ergogenic effects of caffeine ingestion on exercise performance. Nutrients. (2019) 11:732. 10.3390/nu11040732 30934886 PMC6521624

[37] Giovanini de Oliveira Sartori A Vieira da Silva M. Caffeine in Brazil: intake, socioeconomic and demographic determinants, and major dietary sources. Nutrire. (2016) 41:11. 10.1186/s41110-016-0014-x

