# Supplementary material for: Brief Version of Caffeine Expectancy Questionnaire in Brazil
Source: Front Nutr. 2021 Jun 30;8:695385. doi: 10.3389/fnut.2021.695385 (PMC8277974; doi:10.3389/fnut.2021.695385)
Supplement: Supplementary file 1 [file Table_1.docx]

Brief version of Caffeine Expectancy Questionnaire Brazil (B-CaffEQ-BR)

**Supplementary Material**

**Versão curta do Questionário de Expectativa de Efeitos da Cafeína, Brasil (B-CaffEQ-BR)**

Instruções: Estamos interessados em suas crenças sobre os efeitos que a cafeína tem sobre você. Abaixo há uma lista de possíveis efeitos da cafeína quando consumida. Usando a escala como guia, avalia cada afirmação em termos de quanto é PROVÁVEL ou IMPROVÁVEL para esses efeitos como consequência do consumo da cafeína. As possibilidades de respostas são: 1 = Muito improvável; 2 = Improvável; 3 = Um pouco improvável; 4 = Um pouco provável; 5 = Provável; 6 = Muito provável. Baseie suas respostas com base nas fontes de cafeína mais usuais na sua rotina, ou você pode optar por basear suas respostas em “cafeína/café”.

| **Itens** | **Muito improvável** | **Improvável** | **Um pouco improvável** | **Um pouco provável** | **Provável** | **Muito provável** |
| --- | --- | --- | --- | --- | --- | --- |
| 1. Cafeína/café me dá ânimo quando estou cansado(a). | □ | □ | □ | □ | □ | □ |
| 2. Cafeína/café melhora meu desempenho físico. | □ | □ | □ | □ | □ | □ |
| 3. A cafeína/café tira minha fome. | □ | □ | □ | □ | □ | □ |
| 4. Cafeína/café melhora meu humor. | □ | □ | □ | □ | □ | □ |
| 5. Eu fico ansioso(a) quando não tomo cafeína/café. | □ | □ | □ | □ | □ | □ |
| 6. Eu me exercito melhor depois de tomar cafeína/café. | □ | □ | □ | □ | □ | □ |
| 7. Eu sinto muita falta de cafeína/café quando não tomo. | □ | □ | □ | □ | □ | □ |
| 8. Eu não gosto do jeito que eu me sinto após tomar cafeína/café. | □ | □ | □ | □ | □ | □ |
| 9. Tomar cafeína/café a qualquer hora do dia atrapalha o meu sono. | □ | □ | □ | □ | □ | □ |
| 10. Quando tomo cafeína/café fico nervoso(a). | □ | □ | □ | □ | □ | □ |
| 11. Cafeína/café melhora minha concentração. | □ | □ | □ | □ | □ | □ |
| 12. Cafeína/café me faz pular refeições. | □ | □ | □ | □ | □ | □ |
| 13. Tomar cafeína/café na hora de dormir atrapalha meu sono. | □ | □ | □ | □ | □ | □ |
| 14. Cafeína/café me deixa irritado(a). | □ | □ | □ | □ | □ | □ |
| 15. Cafeína/café me faz sentir feliz. | □ | □ | □ | □ | □ | □ |
| 16. Eu não funciono sem tomar cafeína/café. | □ | □ | □ | □ | □ | □ |
| 17. Tomar cafeína/café no final da tarde atrapalha o meu sono. | □ | □ | □ | □ | □ | □ |
| 18. Eu fico mais extrovertido(a) quando tomo cafeína/café. | □ | □ | □ | □ | □ | □ |
| 19. Cafeína/café me ajuda a me exercitar por mais tempo. | □ | □ | □ | □ | □ | □ |
| 20. Cafeína/café me faz sentir com mais energia. | □ | □ | □ | □ | □ | □ |
| 21. Cafeína/café diminui o meu apetite. | □ | □ | □ | □ | □ | □ |
